# Heavy Ion Minibeam Therapy: Side Effects in Normal Brain

**DOI:** 10.3390/cancers13246207

**Published:** 2021-12-09

**Authors:** John G. Eley, Catherine W. Haga, Asaf Keller, Ellis M. Lazenby, Charles Raver, Adam Rusek, Farrokh Avraham Dilmanian, Sunil Krishnan, Jaylyn Waddell

**Affiliations:** 1 Department of Radiation Oncology, Vanderbilt University School of Medicine, Nashville, TN 37232, USA; ellis.lazenby@vumc.org; 2 Department of Radiation Oncology, University of Maryland School of Medicine, Baltimore, MD 21201, USA; CHaga@som.umaryland.edu; 3 Department of Anatomy and Neurobiology, University of Maryland School of Medicine, Baltimore, MD 21201, USA; akeller@som.umaryland.edu (A.K.); CRaver@som.umaryland.edu (C.R.); 4 Brookhaven National Laboratory, Upton, NY 11973, USA; rusek@bnl.gov; 5 NASA Space Radiation Laboratory, Upton, NY 11973, USA; 6 Health Sciences Center, Departments of Radiation Oncology, Radiology, and Neurology, Stony Brook University, Stony Brook, NY 11794, USA; f.dilmanian@stonybrookmedicine.edu; 7 Mayo Clinic Cancer Center, Department of Radiation Oncology, Jacksonville, FL 32224, USA; krishnan.sunil@mayo.edu; 8 Department of Pediatrics, University of Maryland School of Medicine, Baltimore, MD 21201, USA; JWaddell@som.umaryland.edu

**Keywords:** particle therapy, brain cancer, microbeam, pediatric, cognitive, toxicity, brain, CNS, rodent, rat

## Abstract

**Simple Summary:**

Minibeam therapy spares certain normal tissues compared to conventional radiation therapy. Recently, minibeams applied with protons have shown efficacy in rodent models of glioblastoma. In consideration of translation to human therapy, heavier ions such as helium-4, lithium-7, carbon-12, or oxygen-16 would enable the method to reach deeper into the human brain, potentially sparing greater volumes of normal brain; however, biologic uncertainties and potential toxicities of the method are poorly understood, especially for heavy ions. This work explores the cognitive impairments and pathologic changes seen in normal rodent brain at late timepoints after partial-brain minibeam irradiation with heavy ions.

**Abstract:**

The purpose of this work was to investigate whether minibeam therapy with heavy ions might offer improvements of the therapeutic ratio for the treatment of human brain cancers. To assess neurotoxicity, we irradiated normal juvenile rats using 120 MeV lithium-7 ions at an absorbed integral dose of 20 Gy. Beams were configured either as a solid parallel circular beam or as an array of planar parallel minibeams having 300-micron width and 1-mm center-to-center spacing within a circular array. We followed animals for 6 months after treatment and utilized behavioral testing and immunohistochemical studies to investigate the resulting cognitive impairment and chronic pathologic changes. We found both solid-beam therapy and minibeam therapy to result in cognitive impairment compared with sham controls, with no apparent reduction in neurotoxicity using heavy ion minibeams instead of solid beams under the conditions of this study.

## 1. Introduction

Radiotherapy serves a critical role in the treatment of pediatric and adult brain cancers, especially for surgically inoperable sites or for subclinical disease intermingled with critical normal brain tissue. However, especially for pediatric patients, the side effects of radiotherapy seen in brain cancer survivors present serious impairments to normal brain development and can result in persistent quality of life issues such as anxiety, depression, chronic fatigue, academic impairment, impaired executive functioning, and poor social functioning [1,2]. These are in addition to more sensory side effects such as loss of vision, hearing, or sensation, which also affect a large number of brain cancer survivors. Furthermore, since nerve cells are generally post-mitotic cells that do not replicate, neuronal damage is irreparable.

Minibeam therapy is one avenue that might offer reduced toxicity to the normal brain compared with conventional radiation therapy. In minibeam therapy, also called microbeam therapy, beams are typically configured as arrays having submillimeter parallel beamlets and gaps between the beamlets with regular spacing [3,4]. This form of spatial fractionation offers the possibility that progenitor cells surviving in the unirradiated gaps between minibeams have a chance to repopulate and thus repair the organ level damage after radiation exposure [5,6]. Cells directly hit by minibeams presumably die via mechanisms similar to that following conventional solid-beam irradiation, but the unirradiated cells either migrate or proliferate to compensate for this, offering a tissue sparing effect. This effect has been demonstrated since 1959 [7], in a number of animal models using microbeams and minibeams in the size range of tens to hundreds of microns, and it has demonstrated tissue sparing at doses up to several hundreds of Gy in single fraction exposures [5,8,9,10,11]. Despite the preclinical evidence, minibeam therapy has never been translated to human therapy, in part due to the still poorly understood mechanism and dose tolerances relevant for humans and also in part due to the limitations of the radiation modalities used in the majority of these studies.

In response to these limitations and in parallel with advances in particle therapy, it was demonstrated in simulations and experiments [4,12,13,14] that protons could offer the ability to deliver an array of minibeams that would gradually converge into a solid (conventional) proton beam due to multiple Coulomb scattering. In addition, studies have demonstrated the ability of proton minibeam therapy to spare skin [15,16,17]. Recently, the method also showed effectiveness in sparing normal rodent brain and in the treatment of glioblastoma models [18,19,20,21]. Furthermore, heavier ions such as helium-4, lithium-7, carbon-12, and oxygen-16 might treat deeper brain tumors while allowing the spatial minibeam dose distribution to reach deeper depths in the human brain [22,23]. However, the biologic toxicity of heavy ion radiation in normal brain is known to be uncertain, even more so for ion-beams in minibeam configurations.

In this study, we investigated whether minibeam therapy with heavy ions, such as lithium-7, might offer improvements of the therapeutic ratio for the treatment of human brain cancer. Lithium-7 was selected out of other possible ion species due to its elastic scattering properties, which appear optimal for minibeam therapy of deeper human brain tumors [22]. Our hypothesis was that heavy ion minibeam therapy would provide reduced normal brain toxicity in proximal tissues compared to heavy ion solid-beam therapy, when both methods were controlled to deliver an equivalent integral dose. We irradiated rats using lithium-7 ions at an energy and dose relevant for human brain tumor therapy, configured either as solid beams or minibeams. We followed animals for 6 months after treatment and investigated the resulting cognitive impairment and chronic pathologic changes.

## 2. Materials and Methods

### 2.1. Experimental Dosimetry for Lithium-7 Minibeam Therapy

**Figure 1:**
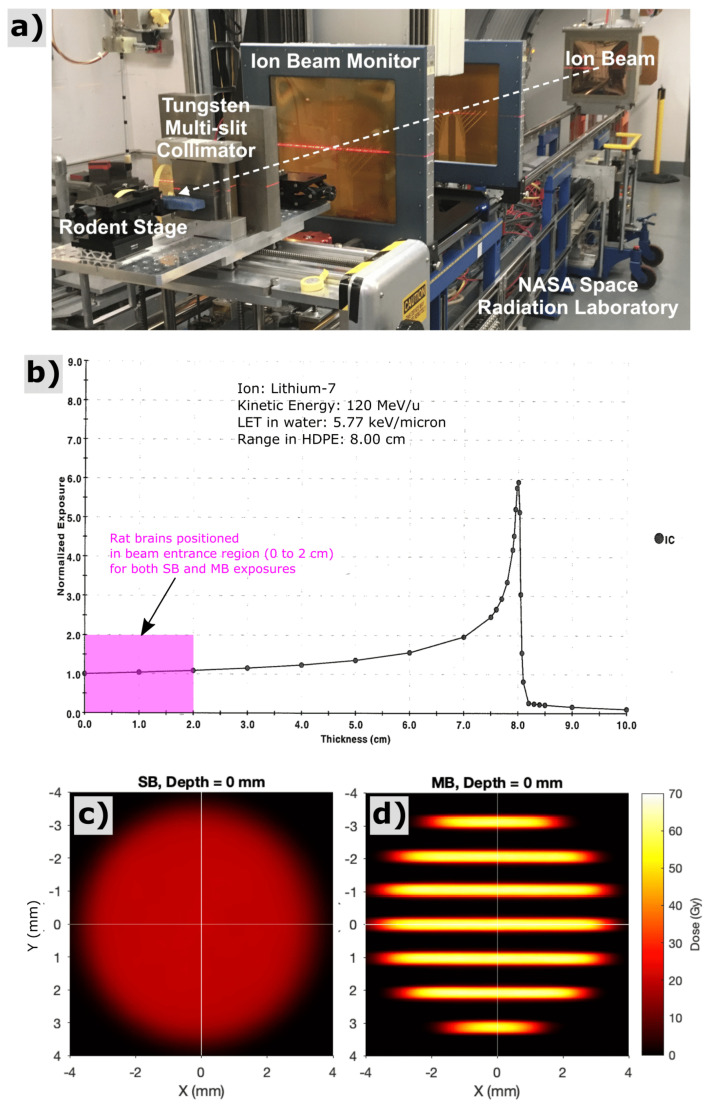
Experimental setup (**a**) for lithium-ion irradiations at the NASA Space Radiation Laboratory (NSRL) at Brookhaven National Laboratory. Bragg-peak measurement (**b**) acquired via the NSRL ionization chamber system for 120 MeV/u lithium-7 ions used in this study. All rodent exposures for this study were performed as shoot-through irradiations in the beam entrance region with depths of 0 to 2 cm (see pink region), far upstream of the Bragg peak. The experiments presented here thus attempt to model radiation-damage to shallow CNS tissues that would lie proximal to a deeper brain tumor. Film measurement of solid-beam (**c**) and minibeam (**d**) spatial dose patterns corresponding to the beam entrance at the surface of the rat’s head, both providing an integral dose over the irradiated region of 20 Gy.

Radiation exposures with lithium-7 ions were performed at the NASA Space Radiation Laboratory (NSRL) facility at Brookhaven National Laboratory (Upton, NY, USA). An energy of 120 MeV/u was used for all exposures, providing a range in tissue of approximately 8 cm and a linear-energy-transfer value of 5.77 keV/μm in water. Exposures were configured, firstly, by collimating the monoenergetic, quasi-parallel, lithium-ion field into a cylindrical solid beam (SB) with 7-mm diameter, which served as our control exposure, and, secondly, by segmenting the SB into an array of parallel planar minibeams (MB) via a 5-cm-thick tungsten multislit collimator. The MB array had 300-micron planar beams with 1-mm center-to-center spacing within the array (see Figure 1). Beams were designed to shoot laterally through the rodent brain, for a partial brain exposure encompassing regions of the hippocampus and prefrontal cortex, regions potentially sensitive to radiation induced cognitive impairment. The SB exposures were calibrated using the NSRL NIST-traceable ionization chamber to provide a mean entrance dose within the circular field of 20 Gy. The MB exposures were calibrated using a secondary parallel-plane ionization chamber that captured the integral ionization across the entire circular fields of both the SB and MB conditions. Based on those ionization chamber measurements, we adjusted the incident fluence of the MB exposure to provide equivalent integral dose of 20 Gy. Relative spatial dose distributions were measured with film (Gafchromic EBT3, Ashland, Covington, KY, USA).

### 2.2. Animal Selection and Definition of Study Arms

Long Evans male juvenile rats were given partial-brain irradiation on post-natal day 27. This age is coarsely equivalent to a human child of 4–7 years of age [24,25,26]. All work was approved by our Institutional Animal Care and Use Committee. Animals (*n* = 18) were allocated randomly into the following study arms: Sham (unirradiated), solid-beam 20 Gy (SB20), and minibeam 20 Gy (MB20). Ketamine/xylazine anesthesia was given intrapertioneally prior to irradiation (50 mg/kg and 6.25 mg/kg, respectively), including to sham animals. After irradiation, animals were housed in pairs in alternating combinations of the study arms, i.e., Sham + SB20, Sham + MB20, and SB20 + MB20, to minimize the potential for housing conditions to affect the study findings. Throughout the study, animals were continually observed for general health, radiation dermatitis, motor deficits, body mass, illness, and humane endpoint criteria. Dermatitis in the irradiated region of the head was assessed using the Douglas and Fowler Scale [27].

### 2.3. Cognitive Testing

Our cognitive tests were selected based on their ability to detect damage to regions critical for complex processing and learning such as the prefrontal cortex and regions of the hippocampus that might potentially be spared damage using heavy ion minibeams. Behavioral testing timelines of 1 month, 3 months, 4 months, and 6 months after irradiation were used to assess the transient and chronic cognitive impairments after irradiation.

Attentional Set Shift (ASST) and Reversal Learning was assessed at 1 and 6 months after irradiation. Rats were placed on food restriction and maintained at 90% ad libitum body weight to motivate choice behavior for a food reward. Our procedure was based on the methods of Birrell and Brown (2000). This task has been adapted from the Wisconsin Card Sort task used to measure frontal lobe function in humans [28]. Briefly, rats were placed in a plexiglass chamber and presented with 2 small bowls. Rats then progressed through a series of discrimination trials in which one of the bowls contained a reward buried in digging media that changed across experimental phases (e.g., beads, aquarium rocks). For each phase, the number of trials to criterion was recorded. The rat was considered to have learned the predictive stimulus when 8 correct choices were made in a row. Rats were tested in 8 phases following an acclimation session in which rats were trained to retrieve the food reward. Phase 1, simple discrimination (SD), proceeded in which the odor cue differed between the bowls (e.g., cumin versus cinnamon) and digging media were the same. The reward was paired with only one odor. Subsequent compound discriminations (CD) were conducted, consisting of two odor cues and two digging media stimuli. Odor continued to be the predictive stimulus dimension and digging media stimuli had to be ignored to choose the reinforced bowl. Intradimensional shifts (IDS) continued to use odor as the predictive dimension, but specific stimuli varied. In reversal phases (R), contingencies were revered after the rat made 8 correct choices. Rats progressed through 3 IDS phases with odor as the predictive dimension to encourage formation of an “attentional set” in which rats attend to the odor cue and ignore the digging media. To confirm formation of the attentional set, an extradimensional shift (EDS), in which the reinforced stimulus changes from odor to digging media, was conducted. Rats that form an attention set require more trials to achieve 8 correct consecutive trials, as more feedback from errors are necessary to switch to the new dimension. Normal rats typically require more trials for criterion during both reversal learning and the EDS phase relative to the phases immediately preceding each. This is referred to as “shift cost.” Analysis is emphasized on reversal phases and on the extradimensional shift phase, as these particularly reveal whether a prior rule was learned and quantify the difficulty to adapt to the new rule.

Performance on the hippocampal-dependent Morris Water Maze was assessed 3 months after irradiation. This test measures spatial learning and memory [29]. A pool with opaque water was surround by white curtains. For training days, distinct cues were attached to the curtains in 4 locations. Rats were first placed in the pool and taught to escape on a visible platform. Then, over 4 days, rats were placed in the pool at 4 different starting locations, while the escape platform remained in the same location. On the 5th day, a probe trial with the escape platform removed was conducted. Rats were then tested in a reversal test, with the escape platform in a novel location. Escape latency was analyzed for each training and reversal trial. For the probe trial, the number of crosses of the previous escape platform region were quantified during the first 15 s of the trial.

Spatial working memory was tested 4 and 6 months after irradiation using a Delayed Non-matching to Place (DNMP) task. Rats were placed in a t-maze and trained to run down the alley and turn either left or right to retrieve a food reward. Once rats readily traversed the maze for reward, either the left or right arm was blocked. The rat was placed in the start position and turned down the open alley for a food reward. The rat was then returned to the start position, and both arms were open. The rat had to turn the opposite direction of the immediately preceding demonstration trial to receive the reward. This initial training was conducted over 2 days. After rats acquired the rule, a working memory delay was imposed. Between each demonstration and test phase of each trial, the rat was confined to the start position of the maze for 30 s. The turn direction was pseudorandom with the condition that one direction was not tested more than 3 times in a row, and an equal number of left and right turns were used in each session. All sessions consisted of 12 trials. The percentage of correct choices was recorded for each session.

### 2.4. Immunohistochemical Study of Chronic Neuroinflammation

To assess potential chronic inflammation, we analyzed histologic features of microglia and astrocytes in the irradiated cortical regions, focusing on ionized calcium-binding adapter molecule 1 (Iba1) and glial fibrillary acidic protein (GFAP), respectively. Following the last 6-month behavioral task, animals were euthanized via IP injection of sodium pentobarbital immediately followed by cardiac perfusion fixation with 10% formalin to preserve the brains for immunohistologic studies. After extraction, brains were stored in 0.05M phosphate buffered saline (PBS), cryoprotected in 30% sucrose, and sectioned into 50-micron sagittal planes including aspects of the irradiated cortex and hippocampus using a sliding microtome (SM2010R, Leica Microsystems, Wetzlar, Germany) fitted with a freezing stage (BFS-40MPA, Physitemp Instruments, Clifton, NJ, USA). Tissues were stained as free-floating sections for dual fluorescence imaging using rabbit anti-Iba1 at 1/5000 final concentration (019-19741, Fuji Wako Chemical USA, Richmond, VA, USA), rabbit anti-GFAP at 1/10,000 final concentration (AB5804, EMD Millipore Corp., Burlington, MA, USA), and Cy3 AffiniPure donkey anti-rabbit at 1/300 final concentration (711-165-152, Jackson ImmunoResearch Laboratories Inc., West Grove, PA, USA). Primary and secondary incubations were preceded by 6, 5-min washes of 0.05M PBS at room temperature, and primary incubations were preceded by a 1-h blocking step with bovine serum albumin. Primary incubation was for 48 h at 4 °C. Secondary incubation duration was for 2 h at room temperature. After staining, sections were wet mounted and imaged using the Olympus FV1000 (Olympus, Tokyo, Japan) and with the Leica Aperio Versa 200 (Leica Biosystems, Buffalo Grove, IL, USA), for confocal and widefield fluorescence imaging, respectively. Anatomic landmarks were used to systematically navigate to cortical regions of interest centered in the radiation exposed regions for each study animal. Microscope settings were identical for all study animals to allow quantitative comparisons between samples. CellProfiler version 3.1.9 [30] was used to analyze Iba1-positive microglia and GFAP-positive astrocyte cell counts and to identify object boundaries used for morphologic analysis.

### 2.5. Statistical Analysis

Prism (Version 8, GraphPad, San Diego, CA, USA) was used for statistical analysis and graphing. Repeated measures analysis of variance (ANOVA) was used for repeated-trial behavioral tests and for time-dependent assessment of radiation dermatitis. One-way ANOVA was used for the probe trial analysis of the Morris Water Maze and for histologic comparisons. A Bonferroni correction was applied to all tests to allow multiple comparisons between Sham, SB20, and MB20. All tests were 2-sided with a = 0.05.

## 3. Results

### 3.1. Experimental Dosimetry for Lithium-7 Minibeam Therapy

**Figure 2:**
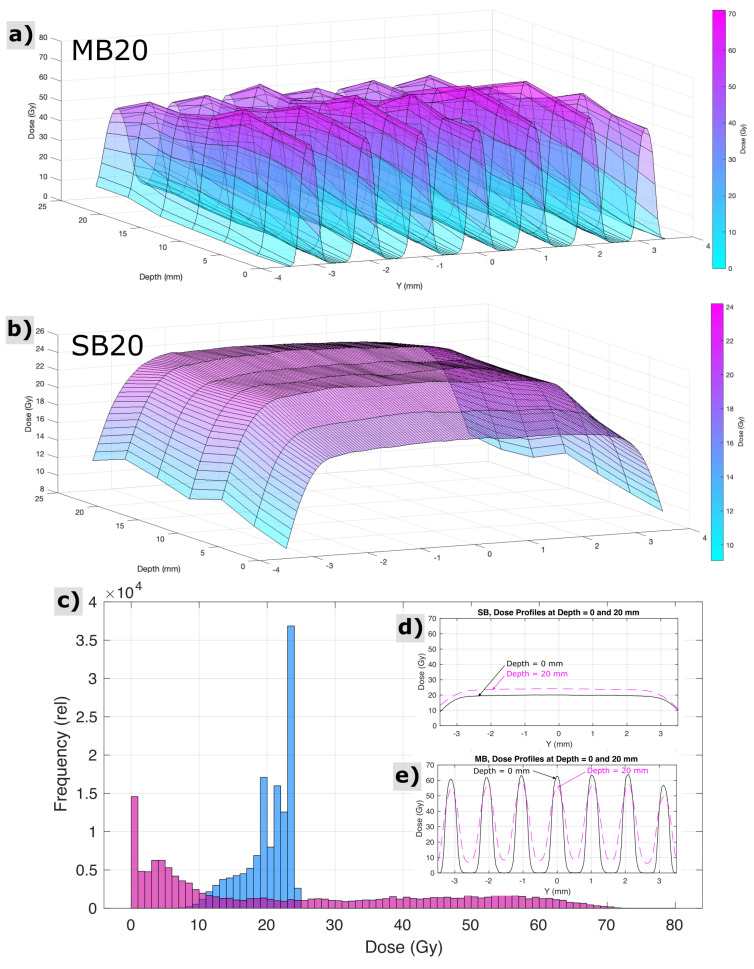
Measured properties of the spatial distribution of dose for minibeam (MB20) and solid beam (SB20) treatment configurations throughout the targeted region of the rat head and brain, i.e., at depths in the animal from 0 to 20 mm and within the circular field boundary (*r* = 3.5 mm). Surface plots indicate the pattern of minibeam peaks and valleys and their variation with depth (**a**) and the comparably homogeneous exposure condition of solid beams (**b**). Histograms (**c**) and cross profiles (**d**,**e**) reveal a different perspective on the same dataset.

For SB20, radiochromic film measurements, cross calibrated to ionization chamber measurements, revealed the dose at the skin entrance to be 20.0 ± 0.5 Gy across the circular beam aperture. For MB20, radiochromic films indicated peak minibeam doses at the skin entrance to be 62.4 ± 1.4 Gy, across the array, and valley minibeam doses across the array to be 2.7 ± 1.0 Gy. The mean peak-to-valley ratio at the entrance for minibeams was 27:1. Figure 2 shows measurements of spatial distributions of dose within the circular field boundary at various depths representative of the rat head, acquired via radiochromic films in a stack of tissue equivalent slabs, spaced every 4 mm in depth and cross-calibrated to ionization chamber measurements. A computed tomography image of 1 age/strain/sex-matched animal (not shown) revealed that the distance from skin to skin, through the rat brain at the targeted region, was approximately 20 mm, and the field boundary for both irradiation conditions had a radius of 3.5 mm. Histograms analyzing the distributions of dose throughout this three-dimensional targeted region are also shown in Figure 2, along with cross profiles of the beam representative of the proximal and distal side of the rat head (0 and 20 mm depths in the tissue-equivalent phantom stack, respectively).

### 3.2. Radiation Dermatitis

**Figure 3:**
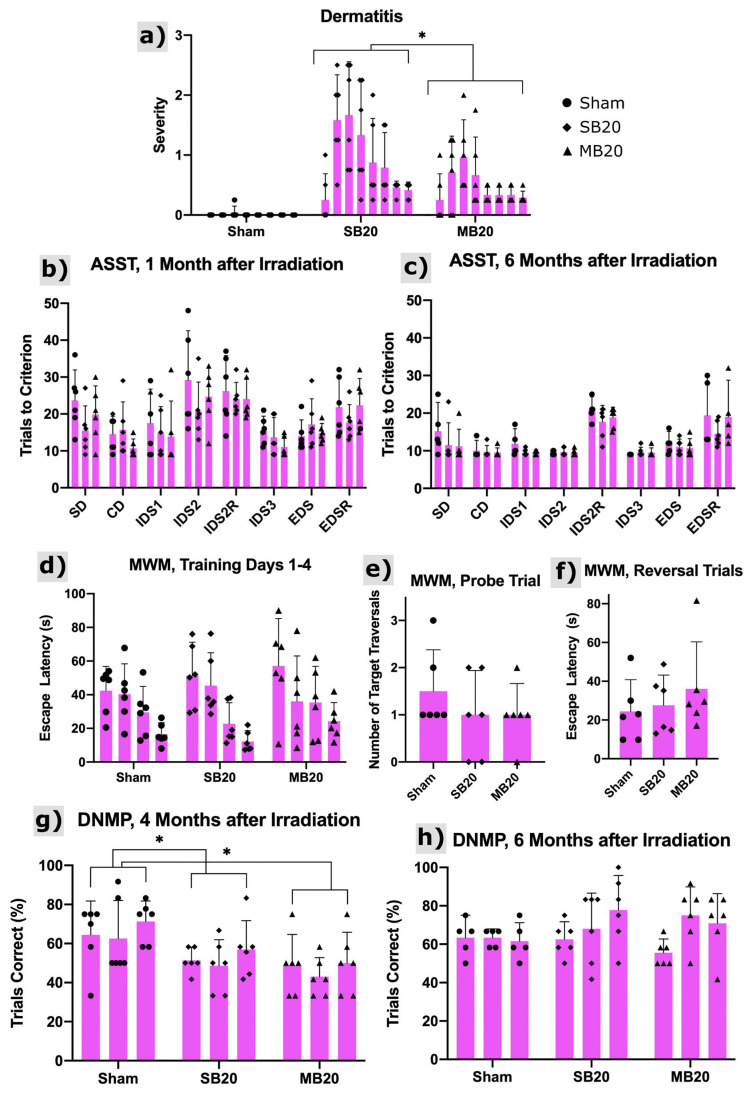
Dermatitis and cognitive testing results. (**a**) Dermatitis severity scores using the Douglas and Fowler Scale from 0 to 8 weeks after irradiation (left to right within arms). Scores reveal onset at 1 to 2 weeks after irradiation with substantial recovery by 8 weeks after irradiation. Minibeams (MB20) significantly (*p* = 0.008, repeated measures analysis of variance, *n* = 6 per arm) reduced the severity of dermatitis compared with solid beams (SB20). Results of the Attentional Set Shifting Task (ASST) at 1 month (**b**) and 6 months (**c**) after irradiation for Sham, SB20, and MB20 study animals. Analysis of the IDS2 Reversal Cost revealed no significant differences between groups but did reveal time as a significant factor (*p* = 0.006), with higher reversal cost at 6 months after irradiation. The EDS Cost and EDS Reversal Cost analyses did not reveal significant differences between study arms. Morris Water Maze (MWM) results at 3 months after irradiation (**d**–**f**). Training trials (**d**) carried out over 4 days (day increasing from left to right within arm) indicate learning for all study groups as the mean escape latency decreased with time. Means and 95% confidence intervals of escape latency over all trial days were 32.2 ± 7.0 s, 32.9 ± 8.5 s, and 38.2 ± 10.6 s for Sham, SB20, and MB20, respectively, i.e., slightly higher for MB20 than SB20 and Sham on average but not significant. For the probe trial (**e**), both irradiated groups showed a lower mean number of target-region traversals than the sham group, indicative of impairment but not significant. For reversal trials (**f**), irradiated groups appeared to be slower to learn the new platform location than the sham group. However, due to the large variances, these differences were also not significant. Delayed Non-matching to Place (DNMP) Task at 4 months (**g**) and 6 months (**h**) after irradiation. Sessions 1–3 for each timepoint increase from left to right within each arm/timepoint. Analysis revealed significantly lower performance on the task for groups SB20 and MB20 compared with Sham at the 4-month timepoint (*p* = 0.05 and 0.01, respectively, repeated measures analysis of variance, *n* = 6 per arm), lowest on average for MB20. No significant differences were seen between groups at the 6-month timepoint. Bars and error bars represent means and 95% confidence intervals. * indicates significant difference with *p* < 0.05.

Figure 3a shows the onset of radiation dermatitis, which was confined to irradiated regions of the head and progressed to moist desquamation for some animals but, later, healed. The severity of skin lesions was significantly lower for MB animals than SB animals (*p* = 0.008).

### 3.3. Cognitive Testing

The results of the Attentional Set Shifting Task (ASST) are shown in Figure 3b,c, with analysis focused on reversals and the extradimensional shift. Reversal of IDS2 6 months after irradiation demonstrates that all groups performed similarly, suggesting that orbitofrontal cortex function is normal irrespective of irradiation treatment [31,32]. No evidence of perseverative responding was detected (IDS2 Reversal). Little reversal cost was observed in any group at the 1-month post-irradiation timepoint. This is likely due to the high number of trials required to complete IDS2. All groups appear to form an attentional set, evidenced by the slightly higher number of trials to solve the extradimensional shift at 6 months post-irradiation. This suggests medial prefrontal cortex function is intact, at least as measured on this non-spatial problem solving task [33,34,35]. No significant differences between study arms were identified.

Results from the Morris Water Maze are shown in Figure 3d–f. Escape latency decreased for all groups across training trials, as evidenced by a significant effect of trial (*p* < 0.0001). Both SB20 and MB20 appear to have higher escape latencies than sham control rats (Figure 3d). This appears to be more persistent in the MB20 group, but group differences failed to reach significance due to the high variability within each group. Though irradiated groups appear to make fewer crossings of the platform location on the probe trial (Figure 3e), this also failed to reach significance due to high variability. Reversal performance (Figure 3f) was similarly suggestive of an effect of irradiation in both SB20 and MB20, but no significant differences were found.

The Delayed Non-matching to Place task assesses spatial working memory. Rats in the SB20 and MB20 group performed poorly in the 4-month post-irradiation test, as shown in Figure 3g. Their performance was significantly different than sham irradiated controls (*p* = 0.05 and *p* = 0.01, respectively) and was worse on average for MB20 than SB20. At the 6-month test (Figure 3h), both the SB20 and MB20 performed as well as controls, indicating that working memory function recovered by this time.

### 3.4. Immunohistochemical Study of Chronic Neuroinflammation

**Figure 4:**
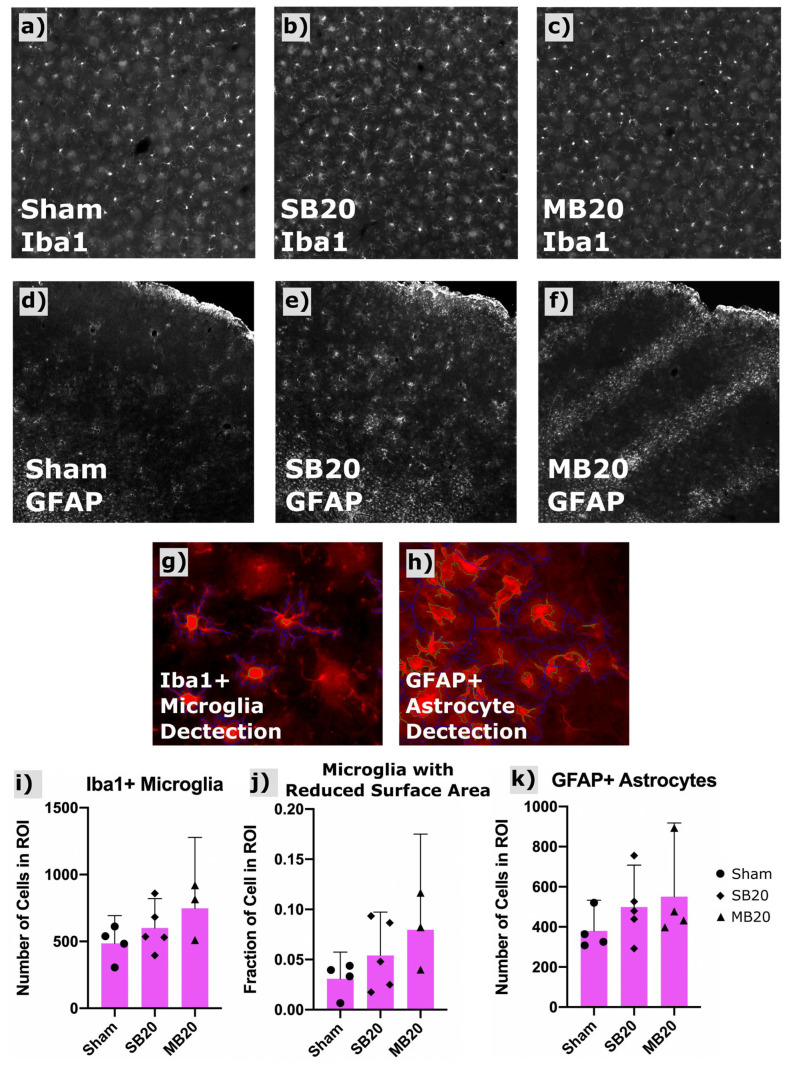
Results of Iba1 and GFAP immunofluorescent staining and automated cell counting in frontal cortex. Representative widefield images of Iba1+ microglia shown for Sham (**a**), SB20 (**b**), and MB20 (**c**) reveal a slightly higher number of labeled cells for irradiated groups. GFAP-labeled fluorescent immunohistologic images for representative animals from Sham (**d**), SB20 (**e**), and MB20 (**f**) study arms. Increased diffuse clusters of GFAP+ astrocytes are seen in SB20 compared with Sham. Localized bands of GFAP+ astrocytes are seen in MB20 corresponding to the dimensions of the minibeam array. Example of automatic detection of primary (green contours) and secondary objects (blue contours) corresponsing to microglia (**g**) and astrocyte (**h**) bodies and processes, respectively. Slightly higher average microglia (**i**) and astrocyte (**k**) cell counts per image were seen in irradiated groups. (**j**) Secondary body perimeter lengths were used to assess the fraction of microglia with a reduced surface area, indicative of activated status, which was higher, on average, for irradiated groups compared with Sham. Bars and error bars represent means and 95% confidence intervals. Datapoints represent cell counts with a region of interest (ROI) in frontal cortex for individual animals.

Figure 4a–c shows a representative widefield image of Iba1-positive microglia. Our image analysis sequence (scripts written for CellProfiler) identified primary objects (microglia bodies) for cell counts and secondary object boundaries used for mapping and morphologic analysis of microglia processes. An increased count of Iba1-positive microglia was seen in irradiated groups, highest for MB20, though these differences were not significant. Activated microglia exhibit shorter process length and more amoeboid morphology than resting microglia, i.e., less total surface area. Therefore, we used secondary object perimeter length (the two-dimensional analogue of surface area) as a surrogate metric for classifying relative numbers of activated versus resting microglia. We quantified the fraction of microglia for each group showing a secondary perimeter length less than a threshold perimeter length, which we defined as those greater than two standard deviations outside (i.e., less than the lower 2.5th percentile) of the sham perimeter-length dataset. The fraction of microglia showing this reduced perimeter length morphology (reduced surface area), indicative of an activated status, was, on average, nearly doubled for SB20 and tripled for MB20, compared with Sham, indicative of chronic neuroinflammation in irradiated groups.

Figure 4d–f show representative confocal images of GFAP-positive astrocytes for each study arm, along with the analysis of astrocyte counts for each treatment group (Figure 4k). Images revealed diffuse clusters of GFAP-positive astrocytes for group SB20 compared with Sham and focal clusters in bands, corresponding to the geometry of the minibeam array, for MB20. Total cell counts indicate higher numbers of GFAP-positive astrocytes for both SB20 and MB20, highest for MB20, though differences were not significant due to large variation between animals.

## 4. Discussion

Our hypothesis was that heavy ion minibeam therapy would cause reduced CNS toxicity compared to heavy ion solid-beam therapy when both methods were controlled to deliver an equivalent integral dose to a hypothetical deeper lying brain tumor. The rationale for this hypothesis was that unirradiated progenitor glial cells in the gaps between minibeams would survive irradiation and proliferate to compensate for any damage to cells in the direct path of minibeams and also that, despite the higher peak dose of minibeams and thus increased cellular radiation damage within the minibeam path, the benefits of this proliferative process would outweigh the benefits of the alternative, standard-of-care approach, namely to use a lower dose solid beam. Overall, the findings of this work do not support our hypothesis and, instead, reveal cognitive impairment and chronic pathologic damage seen in normal rodent brain after minibeam therapy with lithium ions, which was not less severe than that seen after solid-beam therapy, when both methods provided an equivalent integral dose of 20 Gy.

We interpret our findings to mean that sparing of normal tissue between beamlets in a minibeam array does not assure that normal brain function is spared, at least for the doses we studied, the spatial pattern of minibeams we evaluated, and the cognitive function tests and histologic stains we utilized. While we may have been unable to detect subtle differences in cognitive function, no dramatic differences were discernable: both radiation methods produced toxicity. In turn, we conclude that focal sparing of planar tracts of normal brain is unable to compensate for the injury caused by high dose radiation beamlets even though this results in physical sparing of skin erythema, epilation, and desquamation.

This work had several strengths in its design. Firstly, radiation exposures were controlled to minimize free variables. The radiation beam and rodent setup was identical across exposures except for the addition of a multislit collimator, which segmented the beam into an array of minibeams. The integral dose and many other radiation field parameters were identical, such as the beam energy, dose rate, LET, circular field boundary, and rodent positioning table. Animals were randomized according to treatment group and investigators were blind to treatment group for all phases of behavioral testing, immunohistochemistry, and microscopy studies. Lastly, we used high-energy lithium-7 ions, at energies relevant for human brain tumor therapy, which may aid in interpretation of any expected benefits in regard to human therapy.

Our study also had limitations, which were intended by design. Firstly, due to experimental cost restrictions, we did not include x-ray controls. The relatively higher LET of lithium-7 ions is likely accompanied by a higher relative biologic effectiveness (RBE) than x-ray therapy. However, this variable was controlled for in the current study by including the control condition of solid-beam lithium-7 exposures. Thus, the differences in toxicity can be attributed primarily to the spatial patterns and magnitudes of dose throughout the brain. Nevertheless, for this reason, we cannot directly quantify the RBE of this new method. Secondly, we did not yet test the efficacy of heavy ion minibeam therapy for tumor-bearing animals. We intentionally chose to avoid evaluation of treatment toxicity in tumor-bearing animals, since the variability in brain tumor growth between animals would likely increase variability in performance on cognitive tests and lifespan. While this limitation greatly improved our ability to study late cognitive and histologic changes in normal brain with reduced variability between subjects, we acknowledge that the effects of disease progression on brain function and pathology are missing from this work. Another limitation was that we focused immunohistochemical studies on glial cells associated with chronic neuroinflammation. We did not focus on endothelial cell damage, vascular integrity, or neural precursor populations, though we plan to explore those in future studies. Lastly, we acknowledge that larger sample sizes may be needed to distinguish more subtle differences in cognitive toxicity between the methods. Indeed, if we perform a sample-size analysis (α = 0.05, power = 0.8) using the differences in cognitive scores between solid-beam and minibeam treatment using our most sensitive assay (i.e., DNMP task scores at 4 months after irradiation), we can estimate that *n* = 37 animals per arm would be required to distinguish significant differences in cognitive toxicity between the irradiation methods.

In comparison with the existing literature, we found minibeams to substantially reduce the severity of damage to normal skin compared to conventional radiation, which is consistent with previous findings [16,36]. However, in contrast to the work by Prezado et al. [19], we did not find that minibeam therapy spares normal brain. Although we used similar radiation doses, our work is different from theirs in that we used lithium-7 ions, which have a higher LET than proton therapy and, thus, a higher RBE. Another difference was that their work used different biologic endpoints and did not include functional or cognitive testing to assess toxicity. Regarding clinical significance, our data suggest either no significant benefit or even suggest harm could be expected using minibeams instead of solid beams, when integral doses are equal and under the conditions of this study. We again analyzed the means and variances found via the DNMP task at 4-months after irradiation and performed a Number Needed to Treat Analysis (https://clincalc.com/Stats/NNT.aspx, accessed on 29 November 2021). Using that analysis, if we define adverse event incidence as the percentage of animals having a cognitive score that is more than two standard deviations below that of the sham group (i.e., having a score less than 46.8 on DNMP, which was 0/6 for Sham, 1/6 for SB20, and 3/6 for MB20), we can estimate that 1 out of 3 patients could potentially be harmed using heavy ion minibeam therapy instead of solid beam therapy, while 2 out of 3 patients would be expected to have no significant difference in cognitive outcome after minibeam therapy compared with solid beam therapy.

In the future, one application where minibeam therapy might have a clearer therapeutic benefit than seen here would be if we remove the requirement that minibeams provide the same integral dose as solid beams and we remove the requirement that minibeams provide a uniform dose to the tumor. This strategy would avoid the requirement for the higher peak doses of minibeams. We hypothesize that such a geometry might spare immune cells within the minibeam gaps and thus support a robust anti-tumor response that is not possible when a low dose bath covers the entire tumor and surrounding margin. In addition, future studies regarding CNS integrity after heavy-ion minibeam therapy might benefit from investigating potential dysregulation in cerebral blood flow, changes in astrocytic neurovascular coupling, and senescent astrocytes, which have been shown to be critical in radiation-induced cognitive dysfunction and might be uniquely modulated via the spatially fractionated pattern of minibeams [37]. Future experiments are planned to explore these concepts.

## 5. Conclusions

We performed a study of heavy ion minibeam therapy using lithium-7 ions in a rodent model of normal pediatric brain. We compared resulting cognitive impairment and late pathologic changes after minibeam therapy against control exposures of solid-beam therapy having the same integral dose of 20 Gy and against sham controls. We found both solid-beam therapy and minibeam therapy with heavy ions to result in cognitive impairment and chronic neuroinflammation compared with sham therapy, with no apparent reduction in neurologic toxicity using minibeams instead of solid beams under the experimental conditions of this study. Due to their energy-loss and elastic-scattering properties, heavy ions are capable of providing a distinct minibeam spatial pattern of high-dose peaks and low-dose valleys at depths relevant for the treatment of human brain tumors, which cannot be achieved with other external-beam radiation modalities such as x-rays or protons. Despite the evidence of toxicity we found by some metrics, heavy-ion minibeams were generally well tolerated by animals throughout the 6-month study duration, with some cognitive tests showing only minimal differences between minibeam and sham animals and other cognitive tests indicating recovery of cognitive function in treated animals at later timepoints. For these reasons, further investigations of this therapeutic concept are warranted. Overall, our findings support that heavy-ion minibeams provided no robust benefit or harm compared with heavy-ion solid beams, under the experimental conditions of this study.

## 6. Patents

J.G.E., F.A.D., and S.K. report a patent on the application of minibeam therapy with protons and other ions (US 9,962,556 B2).

## Data Availability

Research data are stored in an institutional repository and will be shared upon reasonable request to the corresponding author.
